# A New Clinical Instrument for Estimating the Ambulatory Status after Irradiation for Malignant Spinal Cord Compression

**DOI:** 10.3390/cancers14153827

**Published:** 2022-08-07

**Authors:** Dirk Rades, Ahmed Al-Salool, Christian Staackmann, Florian Cremers, Jon Cacicedo, Darejan Lomidze, Barbara Segedin, Blaz Groselj, Natalia Jankarashvili, Antonio J. Conde-Moreno, Raquel Ciervide, Charlotte Kristiansen, Steven E. Schild

**Affiliations:** 1 Department of Radiation Oncology, University of Lubeck, 23562 Lubeck, Germany; 2 Department of Radiation Oncology, Cruces University Hospital/Biocruces Health Research Institute, 48903 Barakaldo, Spain; 3 Radiation Oncology Department, Tbilisi State Medical University and Ingorokva High Medical Technology University Clinic, Tbilisi 0177, Georgia; 4 Department of Radiotherapy, Institute of Oncology Ljubljana and Faculty of Medicine, University of Ljubljana, 1000 Ljubljana, Slovenia; 5 Department of Radiation Oncology, Acad. F. Todua Medical Center—Research Institute of Clinical Medicine, Tbilisi 0112, Georgia; 6 Department of Radiation Oncology, University and Polytechnic Hospital La Fe, 46026 Valencia, Spain; 7 Department of Radiation Oncology, University Hospital HM Hospitales, Sanchinarro, 28050 Madrid, Spain; 8 Department of Oncology, Vejle Hospital, University Hospital of Southern Denmark, 7100 Vejle, Denmark; 9 Department of Radiation Oncology, Mayo Clinic, Scottsdale, AZ 85259, USA

**Keywords:** malignant spinal cord compression, irradiation, ambulatory status, prognostic instrument, prospective trials

## Abstract

**Simple Summary:**

Since 2005, upfront surgery has been increasingly used in addition to radiotherapy for patients with malignant spinal cord compression (MSCC). As spinal surgery includes significant risks, careful patient selection is crucial. Individual risks and benefits should be considered when choosing an optimal treatment strategy. Benefits include preserving or regaining a patient’s ambulatory function. To facilitate the decision pro or contra upfront surgery, a new prognostic score was developed to predict ambulatory status after radiotherapy alone. This clinical score was created from data of patients previously treated in prospective trials. It includes three prognostic groups (17–21, 22–31, and 32–37 points) with post-radiotherapy ambulatory rates of 10%, 65%, and 97%, respectively. Patients of the 32–37 points group may not require upfront surgery. The new instrument achieved very high accuracy in predicting post-radiotherapy ambulatory and non-ambulatory status and was more precise than a previous prognostic score in predicting non-ambulatory status.

**Abstract:**

Estimating post-treatment ambulatory status can improve treatment personalization of patients irradiated for malignant spinal cord compression (MSCC). A new clinical score was developed from data of 283 patients treated with radiotherapy alone in prospective trials. Radiotherapy regimen, age, gender, tumor type, interval from tumor diagnosis to MSCC, number of affected vertebrae, other bone metastases, visceral metastases, time developing motor deficits, ambulatory status, performance score, sensory deficits, and sphincter dysfunction were evaluated. For factors with prognostic relevance in the multivariable logistic regression model after backward stepwise variable selection, scoring points were calculated (post-radiotherapy ambulatory rate in % divided by 10) and added for each patient. Four factors (primary tumor type, sensory deficits, sphincter dysfunction, ambulatory status) were used for the instrument that includes three prognostic groups (17–21, 22–31, and 32–37 points). Post-radiotherapy ambulatory rates were 10%, 65%, and 97%, respectively, and 2-year local control rates were 100%, 75%, and 88%, respectively. Positive predictive values to predict ambulatory and non-ambulatory status were 97% and 90% using the new score, and 98% and 79% using the previous instrument. The new score appeared more precise in predicting non-ambulatory status. Since patients with 32–37 points had high post-radiotherapy ambulatory and local control rates, they may not require surgery.

## 1. Introduction

Malignant spinal cord compression (MSCC) is considered an emergency that, depending on the type of primary tumor, occurs in up to 10% of adult cancer patients [1,2,3]. For many years, radiotherapy alone was the standard treatment for these patients. In 2005, a randomized trial of 101 patients showed that in selected patients with comparably good performance status and an expected lifespan of at least 3 months, treatment outcomes were improved with addition of upfront decompressive surgery plus stabilization [4]. In 2010, a matched-pair study of 324 less selected patients was presented [5]. Data from 108 patients treated with surgery plus radiotherapy were matched (1:2) to 216 patients treated with radiotherapy alone for eleven patient- and disease-related characteristics. Outcomes were not significantly different between both treatment groups. Recently, another matched-pair study was reported that included data from prospectively evaluated patients [6]. In this study, upfront surgery followed by radiotherapy resulted in significantly better early improvement of motor function and non-significantly better long-term control of MSCC. However, 36.7% of the patients receiving surgery did not complete their radiotherapy course as planned. It was concluded that patients scheduled for upfront surgery need to be carefully selected. In the randomized trial from 2005, surgery-related complications occurred in 12% of patients after primary treatment and in 40% of patients after salvage treatment [4]. Thus, it would be helpful if neurosurgeons and radiation oncologists were able to estimate the individual benefits and risks of adding surgery to radiotherapy for each patient. This estimation can be supported and facilitated by prognostic instruments. An important goal of the treatment of MSCC is to preserve or regain ambulatory status, which should be considered when making the decision pro or contra upfront surgery.

Therefore, a prognostic instrument (“ambulatory score”) was created in 2008 from the data of 2096 retrospectively evaluated patients treated with radiotherapy alone between 1996 and 2007 [7]. However, since the development of this instrument, treatment concepts have changed and upfront surgery has become more popular, which may reduce its predictive value. Thus, a new prognostic tool may be necessary that can estimate an individual patient’s probability to be ambulatory after radiotherapy alone. In the present study, a new ambulatory score was created from patients treated with radiotherapy alone in prospective trials since 2010 [8,9,10,11]. In addition, the new clinical score was compared to the previous score for correct prediction of post-radiotherapy ambulatory and non-ambulatory status.

## 2. Results

In the entire cohort, 74% of the patients were ambulatory at 1 month following radiotherapy. In no patient did the ambulatory status change between follow up at 1 month and at 3 months. On univariable analyses (Table 1), post-radiotherapy ambulatory status was significantly associated with Eastern Cooperative Oncology Group performance score (ECOG-PS) of 1–2 (*p* < 0.0001), favorable primary tumor type (*p* = 0.002), affection of only 1–2 vertebrae by MSCC (*p* = 0.044), time developing motor deficits >7 days (*p* < 0.0001), pre-radiotherapy ambulatory status (*p* < 0.0001), absence of sensory deficits (*p* < 0.0001), absence of sphincter dysfunction (*p* < 0.0001), and higher doses of radiotherapy given as equivalent doses in 2Gy fractions (*p* = 0.002). ECOG-PS was not included in the multivariate analyses, because ambulatory status and ECOG-PS were confounding variables. Non-ambulatory patients had an ECOG-PS of 3–4, and most ambulatory patients had an ECOG-PS of 1–2.

In the initial full logistic regression model considering all factors with *p* ≤ 0.25 on univariate analyses, pre-radiotherapy ambulatory status (*p* < 0.001), sensory deficits (*p* = 0.005), and sphincter dysfunction (*p* = 0.017) were significant. Primary tumor type (*p* = 0.15), number of vertebrae affected by MSCC (*p* = 0.83), visceral metastases (*p* = 0.28), time developing motor deficits (*p* = 0.15), and dose of radiotherapy (*p* = 0.57) did not achieve significance. After applying a backward stepwise variable selection technique to the initial model (Table 2), primary tumor type (*p* = 0.036), pre-radiotherapy ambulatory status (*p* < 0.0001), sensory deficits (*p* = 0.004), and sphincter dysfunction (*p* = 0.008) remained in the parsimonious model. Pairwise interactions between these four factors were not detected (each *p*-value > 0.50). For internal validation, bootstrapping with 1000 replications was used. While the naïve C-statistic was 0.93, the “optimism corrected” C-statistic accounting for overfitting was still 0.91, confirming the good predictive performance of the model.

**Figure 1:**
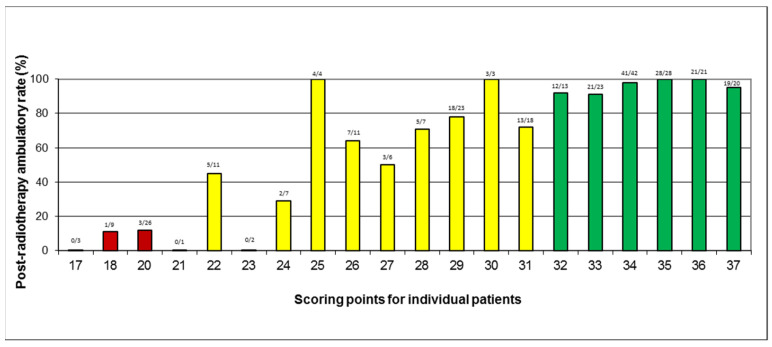
Scoring points for individual patients and post-radiotherapy ambulatory rates (in %). Colours represent the prognostic groups, i.e., red = least favorable group, yellow = intermediate group, and green = most favorable group.

**Figure 2:**
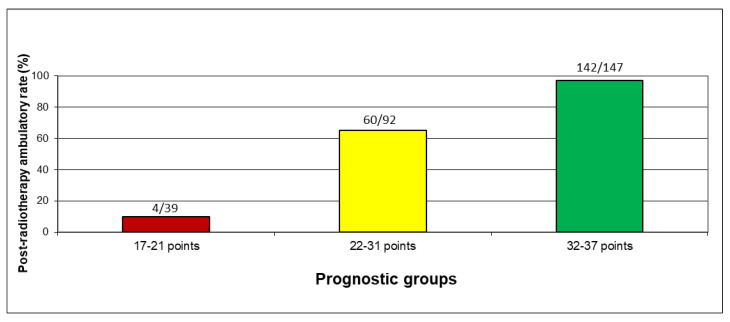
Post-radiotherapy ambulatory rates (in %) of the three prognostic groups. Colours represent the prognostic groups, i.e., red= least favorable group, yellow = intermediate group, and green = most favorable group.

Data for all four factors used for developing the ambulatory score (primary tumor type, sensory deficits, sphincter dysfunction, pre-radiotherapy ambulatory status) were available in 278 patients (98%). After summing the scoring points of the four factors (Table 3) individually for each patient, total patient scores between 17 and 37 points were obtained. The corresponding post-radiotherapy ambulatory rates are shown in Figure 1. Based on these rates, three prognostic groups were formed, namely 17–21 points, 22–31 points, and 32–37 points. The post-radiotherapy ambulatory rates of these groups were 10% (4 of 38 patients), 65% (60 of 92 patients), and 97% (142 of 147 patients), respectively (*p* < 0.0001, Chi-square test) (Figure 2). The differences between 17–21 points and 22–31 points (*p* < 0.0001, Fisher’s exact test) and between 22–31 points and 32–37 points (*p* < 0.0001, Chi square test) were highly significant. Of the patients who were ambulatory following radiotherapy, 0 of 4 patients (0%) in the 17–21 points group, 25 of 60 patients (42%) in the 22–31 points group, and 104 of 142 patients (73%) in the 32–37 points group, respectively, were able to walk without aid.

In the three groups, the rates of local control of MSCC were 100%, 86%, and 91%, respectively, at 1 year, and 100%, 75%, and 88%, respectively, at 2 years (*p* = 0.54). Survival rates were 19%, 26%, and 54%, respectively, at 1 year, and 13%, 13%, and 40%, respectively, at 2 years (*p* < 0.001). The positive predictive values (PPVs) of the new instrument to correctly predict ambulatory status and non-ambulatory status were 97% and 90%, respectively. When using the previous instrument from 2008, the PPVs were 98% and 79%, respectively [5].

## 3. Discussion

For many years, radiotherapy alone was considered the standard treatment for MSCC. Since 2005, when a randomized demonstrated better treatment outcomes for selected patients when upfront surgery was added to radiotherapy, this combined approach has become increasingly popular [4]. However, the question remains as to who can benefit from the addition of surgery. To be eligible for the trial from 2005, patients were required to have a good performance score to tolerate spinal surgery, an expected survival time of 3 months or longer, and MSCC restricted to a single area of the spine (several contiguous segments allowed) [4]. Furthermore, patients should not have been completely paraplegic for more than 48 h and should not have MSCC from very radiosensitive tumors such as lymphoma, leukemia, myeloma, and germ-cell tumors. Moreover, in a matched-pair study, patients with MSCC from unfavorable tumors such as non-small-cell lung cancer, cancer of unknown primary, renal cell carcinoma, or colorectal cancers appeared to benefit from decompressive surgery plus stabilization in addition to radiotherapy [12]. Two other matched-pair studies not limited to patients with MSCC from unfavorable tumors produced conflicting results [5,6]. In the first of these studies, 108 patients who received surgery followed by radiotherapy were retrospectively matched (1:2) to 216 patients irradiated without upfront surgery [5]. Motor function improved in 27% and 26% of patients, respectively (*p* = 0.92), and post-treatment ambulatory rates were 69% and 68%, respectively (*p* = 0.99). Local control and survival rates at 1 year were 90% vs. 91% (*p* = 0.48) and 47% vs. 40% (*p* = 0.50), respectively. In the surgery plus radiotherapy group, 11% of the patients experienced surgery-related complications. In the second of these matched-pair studies that included data from prospectively evaluated patients, the addition of upfront surgery resulted in a significantly higher rate of early improvement of motor function (39.2% vs. 21.5%, *p* = 0.015) [6]. No significant differences were found for post-treatment ambulatory rates (59.5% vs. 67.1%, *p* = 0.32) and survival (*p* = 0.51). The 1-year local control rate of MSCC was higher after surgery plus radiotherapy than after radiotherapy alone (90.1% vs. 76.2%), although this difference was not significant. However, more than one-third of the patients scheduled for the combined treatment did not complete the planned radiotherapy course, mainly due to a decreased performance status following surgery [6].

These results demonstrate that it is very important to carefully evaluate a patient before assigning him or her to upfront surgery. To facilitate this evaluation, it would be helpful to estimate the risks and benefits (i.e., improved treatment results) of the additional surgery. Benefits include several endpoints, of which the treatment effect on the patient’s ambulatory status is of particular importance. It would be helpful to be able to estimate an individual patient’s probability to be ambulatory following radiotherapy alone. In case of a high probability of being ambulatory, upfront surgery may not be necessary. In contrast, if the probability is low, patients very likely benefit from addition of surgery to radiotherapy. Estimation of post-radiotherapy ambulatory status would be considerably facilitated if a corresponding prognostic instrument was available.

Such an instrument was developed in 2008 using retrospective data of 2096 patients receiving radiotherapy alone without upfront surgery [7]. Based on five independent predictors of post-radiotherapy ambulatory status (primary tumor type, interval between tumor diagnosis and MSCC, presence of visceral metastases, pre-radiotherapy motor function and time developing motor deficits) five prognostic groups were designed, of 21–28, 29–31, 32–34, 35–37, and 38–44 points [7]. In 2011, this score was validated and simplified by reducing the number of prognostic groups from five to three, i.e., 21–28, 29–37 and 38–44 points [13]. However, because upfront surgery has been increasingly used after the publication of the randomized trial in 2005, the predictive value of this prognostic instrument likely has decreased [4]. Thus, the time appeared ripe for a new scoring instrument based on data from patients with MSCC treated with radiotherapy alone after the publication of the randomized trial. Therefore, the present study was conducted, which aimed to fill this gap and produce a new scoring instrument for estimating post-radiotherapy ambulatory status. To overcome the shortcomings of the previous score, only patients treated 2010 and later were considered for the present study. Moreover, the patients were treated within a prospective phase II or phase III trial [8,9,10,11].

In the present study, four prognostic factors that were associated with post-radiotherapy ambulatory status in the parsimonious multivariable logistic regression model after backward stepwise variable selection were used to develop the new score. Primary tumor type and pre-radiotherapy ambulatory status were already identified as significant predictors of post-radiotherapy ambulatory status when the previous scoring tool was developed [7]. Sphincter dysfunction is generally accepted as a good indication for upfront surgery, since the outcomes after radiotherapy are often not satisfying [1,2,14,15]. However, surgery (and anesthesia) cannot always be performed, particularly if the patients have a poor performance score or significant comorbidities. Moreover, some patients refuse surgery in a palliative situation like MSCC. The prognostic impact of pre-radiotherapy sensory deficits on post-treatment ambulatory status was not previously identified. In a study by Helweg-Larsen et al., the median time of sensory disturbances prior to the diagnosis of MSCC was shorter than for radicular pain and motor deficits [16]. One may speculate whether the later occurrence of sensory deficits may be an indicator of a more advanced stage of MSCC, which is more difficult to treat.

In the present study, three prognostic groups were designed (17–21, 22–31 and 32–37 points) with post-radiotherapy ambulatory rates of 10%, 65%, and 97%, respectively. The differences between 17–21 and 22–31 points and between 22–31 and 32–37 points were highly significant. The PPV for the new instrument to correctly predict non-ambulatory status was higher than for the previous scoring tool (90% vs. 79%) [7,13]. The PPVs to correctly predict ambulatory status were almost identical and very high for both instruments, namely 97% for the new score and 98% for the previous score, respectively [7,13]. Thus, when considering both PPVs, the new score appeared more precise. Moreover, in the present study, long-term local control of MSCC was achieved in all three groups, i.e., also in patients of the 32–37 points group. Therefore, patients of this group may not require upfront surgery, and omission of this additional treatment, which bears a risk of complications, should be seriously considered for these patients [4,8,12]. In contrast, patients of the 17–21 points group very likely benefit from upfront surgery, since only very few patients were ambulatory following radiotherapy alone. Although the majority of the patients of the 22–31 points group were ambulatory after radiotherapy, many patients of this group may benefit from upfront surgery. Particularly for this group, other factors including the eligibility criteria of the randomized trial from 2005 need to be considered for optimally tailoring the treatment to an individual patient’s situation [4]. However, when following these recommendations, the retrospective nature of the present study should be considered. Despite the fact that the data used to develop the new prognostic instrument were obtained from prospective phase II or phase III trials, the risk of a hidden bias still exists.

The new clinical score should not be used alone but in addition to other instruments that have been developed to facilitate the decision pro or contra upfront surgery. One existing instrument is the Bilsky classification [17]. It describes the degree of epidural spinal cord compression (six-point grading system) based on T2-weighed magnetic resonance images. However, the Bilsky classification does not consider clinical aspects such as neurological deficits, which are very important—from the patient’s perspective, as well. Moreover, our new clinical score is easy-to-use, which is an advantage in an oncological emergency situation like MSCC. Another existing instrument is the Spinal Instability Neoplastic Score (SINS), which is based on patient symptoms and radiographic criteria including location of the vertebral metastases, pain, type of bone lesion, radiographic spinal alignment, extent of vertebral body collapse, and posterolateral involvement of spinal elements [18,19]. The SINS was designed to support physicians to identify patients who likely benefit from upfront surgery by estimating spinal instability. However, it does not predict neurological outcomes like our new clinical score [20]. Therefore, the SINS and our new ambulatory score complement each other and should both be used for optimal decision making regarding upfront decompressive surgery.

## 4. Materials and Methods

Data of 283 patients treated with radiotherapy alone (i.e., without upfront decompressive surgery) for MSCC in one of three prospective trials between 2010 and 2022 were re-evaluated [8,9,10,11]. The present study was approved by the ethics committee of the University of Lübeck in its original form on 5 December 2021 (reference 21–478) and in amended form on 11 February 2022. Patients had motor deficits of at least one leg for 0–30 days caused by MSCC of the thoracic and/or lumbar spine. Radiotherapy was performed with conventional radiotherapy (*n* = 193) or high-precision radiotherapy including volumetric modulated arc therapy (*n* = 78), intensity-modulated radiation therapy (*n* = 10) and stereotactic body radiation therapy (*n* = 2). Dose-fractionation regimens included 5 × 4 Gy, 5 × 5 Gy, 10 × 3 Gy, 12 × 2.633 Gy plus 3 × 2.333 Gy, 15 × 2.633 Gy, 15 × 2.333 Gy plus 3 × 2.0 Gy, and 18 × 2.333 Gy. The corresponding equivalent doses in 2 Gy fractions (*α/β* ratio of 10 Gy for tumor cell kill) were 23.3 Gy, 31.3 Gy, 32.5 Gy, 41.4 Gy, 42.0 Gy, and 43.2 Gy, respectively. Patients previously treated in the randomized SCORE-2 trial received 5 × 4 Gy or 10 × 3 Gy, patients treated in the PRE-MODE trial 5 × 5 Gy, and patients treated in the RAMSES-01 trial equivalent doses in 2Gy fractions > 40 Gy [8,9,10,11]. Thus, the dose–fractionation regimen was strongly correlated with the trial. Dose–fractionation of radiotherapy plus 12 patient- and disease-related factors were analyzed for associations with ambulatory status (ambulatory vs. not ambulatory) at 1 month and 3 months following radiotherapy. In accordance with the definition of the primary endpoint in the randomized SCORAD trial and secondary endpoints in our previous trials, post-treatment ambulatory status was defined as being ambulatory without or with aid [8,9,21].

The 12 factors included age (≤67 vs. >67 years, median age = 67 years), gender (female vs. male), ECOG-PS (1–2 vs. 3–4), primary tumor type (*n* ≥ 10, namely breast cancer vs. prostate cancer vs. myeloma/lymphoma vs. non-small-cell lung cancer vs. small-cell lung cancer vs. cancer of unknown primary vs. renal cell carcinoma vs. colorectal cancer vs. other tumor types), interval between tumor diagnosis and MSCC (≤6 vs. >6 months, median interval = 6 months), number of vertebrae affected by MSCC (1–2 vs. ≥3, median number = 2), f visceral metastases at the start of radiotherapy (no vs. yes), other bone metastases at the start of radiotherapy (no vs. yes), time developing motor deficits prior to the start of radiotherapy (faster (0–7 days) vs. slower (>7 days), pre-radiotherapy ambulatory status (ambulatory without aid vs. ambulatory with aid vs. not ambulatory), pre-radiotherapy sensory deficits (no vs. yes), and pre-radiotherapy sphincter dysfunction (no vs. yes) (Table 4). The starting point of motor deficits for the factor “time developing motor deficits” was calculated from the onset of patient-reported symptoms. For the factor “sensory deficits”, any degree of deficits according to patient-reported symptoms was considered.

For univariable analyses, the Chi-square test was used; *p*-values of <0.05 were considered indicative of significance. The initial multivariable analysis was performed with a logistic regression model including those factors with a *p*-value of ≤0.25. Subsequently, a backward stepwise selection was applied to identify the parsimonious model restricted to the most relevant prognostic factors; a significance level of 0.1 for a variable to stay in the model was chosen. For internal model validation, bootstrap-corrected concordance C-statistic was used to evaluate the predictive model performance. The C-statistic describes the association between the predicted probabilities and observed response. In binary outcomes, the C-statistic is equivalent to the area under the receiver operating curve and represents the probability that a patient with an outcome is given a higher probability by the model than a random patient without the outcome. Typically, C-index values that exceeded 0.6 were suggestive of a reasonable estimation. These statistical analyses were performed with SAS 9.4 (SAS Institute Inc., Cary, NC, USA).

For each factor in the parsimonious regression model, scoring points were calculated by dividing the post-radiotherapy ambulatory rates (given in %) by 10. The scoring points of these factors were added for each patient. Based on the post-radiotherapy ambulatory rates, prognostic groups were designed. In addition to post-radiotherapy ambulatory status, the prognostic groups were analyzed with respect to local control of MSCC (freedom from an in-field recurrence of MSCC in the irradiated parts of the spine) and survival using the Kaplan–Meier method and the log-rank test. Local control and survival were calculated from the last day of the radiotherapy course (Table 4).

In addition, the new prognostic instrument was compared to the previous score [7] for correct prediction (positive predictive value, PPV) of post-radiotherapy ambulatory status (best prognostic group, i.e., 32–37 points) and non-ambulatory status (worst prognostic groups, i.e., 17–21 points).

The PPV to correctly predict ambulatory status in best prognostic group was calculated as follows:

*PPV = (ambulatory patients/(ambulatory patients + non-ambulatory patients)) × 100*

The PPV to correctly predict non-ambulatory status in worst prognostic group was calculated as follows:

*PPV = (non-ambulatory patients/(non-ambulatory patients + ambulatory patients)) × 100*

## 5. Conclusions

In conclusion, the new clinical score achieved very high accuracy in predicting post-radiotherapy ambulatory and non-ambulatory status. It appeared more precise than the previous score in predicting non-ambulatory status. Depending on their survival prognoses, patients with 17–21 points and many patients with 22–31 points likely benefit from upfront surgery. Since patients of the 32–37 points group had very high rates of both post-radiotherapy ambulatory status and long-term local control of MSCC, they may not require surgery in addition to radiotherapy. When aiming to follow these suggestions, the retrospective nature of the current study should be kept in mind. Although the data for creating the new prognostic instrument came from prospective trials, the risk of a hidden bias could not be entirely excluded. This clinical score should be used in addition to existing instruments such as the SINS to properly identify patients who benefit from upfront decompressive surgery.

## Figures and Tables

**Table 1:** Univariable analyses: Ambulatory rates following radiotherapy. The *p*-values were obtained using the Chi-square test.

Factor	Post-Radiotherapy Ambulatory Rate (%)	*p*-Value
Age		
≤67 years (*n* = 146)	73	0.82
>67 years (*n* = 137)	75	
Gender		
Female (*n* = 108)	75	0.73
Male (*n* = 175)	73	
ECOG performance score		
1–2 (*n* = 119)	95	**<0.0001**
3–4 (*n* = 164)	59	
Primary tumor type		
Breast cancer (*n* = 52)	87	**0.002**
Prostate cancer (*n* = 49)	74	
Myeloma/lymphoma (*n* = 30)	87	
Non-small-cell lung cancer (*n* = 59)	73	
Small-cell lung cancer (*n* = 13)	92	
Cancer of unknown primary (*n* = 23)	65	
Renal cell carcinoma (*n* = 11)	82	
Colorectal cancer (*n* = 10)	40	
Other tumor types (*n* = 36)	53	
Interval tumor diagnosis to MSCC		
≤6 months (*n* = 143)	75	0.71
>6 months (*n* = 140)	73	
Number of affected vertebrae		
1–2 (*n* = 162)	78	**0.044**
≥3 (*n* = 121)	68	
Visceral metastases		
No (*n* = 110)	78	0.19
Yes (*n* = 173)	71	
Other bone metastases		
No (*n* = 44)	68	0.35
Yes (*n* = 239)	75	
Time developing motor deficits		
0–7 days (*n* = 99)	58	**<0.0001**
>7 days (*n* = 184)	83	
Pre-radiotherapy ambulatory status		
Ambulatory without aid (*n* = 82)	99	**<0.0001**
Ambulatory with aid (*n* = 99)	91	
Not ambulatory (*n* = 102)	37	
Pre-radiotherapy sensory deficits *		
No (*n* = 144)	90	**<0.0001**
Yes (*n* = 135)	58	
Pre-radiotherapy sphincter dysfunction **		
No (*n* = 219)	86	**<0.0001**
Yes (*n* = 63)	32	
Radiotherapy regimen		
5 × 4 Gy (*n* = 97)	68	**0.002**
10 × 3/5 × 5 Gy (*n* = 136)	71	
>40 Gy (*n* = 50)	94	

ECOG: Eastern Cooperative Oncology Group; * unknown: *n* = 4; ** unknown: *n* = 1; MSCC: malignant spinal cord compression; bold *p*-values are significant.

**Table 2:** Results of the multivariable analysis (odds ratio estimates and Wald confidence intervals) regarding associations with post-radiotherapy ambulatory status after applying a logistic regression model with backward stepwise variable selection (parsimonious model).

Factor	Odds Ratio Estimate	95% Confidence Limits	*p*-Value
Primary Tumor Type			
Breast cancer vs. NSCLC	3.00	0.71–12.62	**0.036**
Prostate cancer vs. NSCLC	7.57	1.78–32.12	
Myeloma/lymphoma vs. NSCLC	21.35	3.53–129.11	
Small-cell lung cancer vs. NSCLC	3.28	0.29–36.84	
Cancer of unknown primary vs. NSCLC	4.13	0.84–20.43	
Renal cell carcinoma vs. NSCLC	2.99	0.26–34.78	
Colorectal cancer vs. NSCLC	0.85	0.09–7.60	
Other tumor types vs. NSCLC	1.48	0.39–5.67	
Pre-radiotherapy ambulatory status			
Ambulatory without aid vs. no ambulatory	102.20	12.18–857.32	**<0.0001**
Ambulatory with aid vs. no ambulatory	12.23	4.72–31.72	
Pre-radiotherapy sensory deficits			
No vs. yes	4.14	1.56–11.00	**0.004**
Pre-radiotherapy sphincter dysfunction			
No vs. yes	4.07	1.44–11.46	**0.008**

NSCLC: non-small lung cancer (largest subgroup); bold *p*-values are significant.

**Table 3:** Scoring points assigned to the prognostic factors included in the scoring instrument.

Factor	Post-Radiotherapy Ambulatory Rate (%)	Scoring Points
Primary Tumor Type		
Breast cancer	87	9
Prostate cancer	74	7
Myeloma/lymphoma	87	9
Non-small-cell lung cancer	73	7
Small-cell lung cancer	92	9
Cancer of unknown primary	65	7
Renal cell carcinoma	82	8
Colorectal cancer	40	4
Other tumor types	53	5
Pre-radiotherapy ambulatory status		
Ambulatory without aid	99	10
Ambulatory with aid	91	9
Not ambulatory	37	4
Pre-radiotherapy sensory deficits		
No	90	9
Yes	58	6
Pre-radiotherapy sphincter dysfunction		
No	86	9
Yes	32	3

**Table 4:** Potential prognostic factors evaluated for post-radiotherapy ambulatory status.

Factor	Number of Patients	Proportion (%)
Age		
≤67 years	146	52
>67 years	137	48
Gender		
Female	108	38
Male	175	62
ECOG performance score		
1–2	119	42
3–4	164	58
Primary tumor type		
Breast cancer	52	18
Prostate cancer	49	17
Myeloma/lymphoma	30	11
Non-small-cell lung cancer	59	21
Small-cell lung cancer	13	5
Cancer of unknown primary	23	8
Renal cell carcinoma	11	4
Colorectal cancer	10	4
Other tumor types	36	13
Interval tumor diagnosis to MSCC		
≤6 months	143	51
>6 months	140	49
Number of affected vertebrae		
1–2	162	57
≥3	121	43
Visceral metastases		
No	110	39
Yes	173	61
Other bone metastases		
No	44	16
Yes	239	84
Time developing motor deficits		
0–7 days	99	35
>7 days	184	65
Pre-radiotherapy ambulatory status		
Ambulatory without aid	82	29
Ambulatory with aid	99	35
Not ambulatory	102	36
Pre-radiotherapy sensory deficits		
No	144	51
Yes	135	48
Unknown	4	1
Pre-radiotherapy sphincter dysfunction		
No	219	77
Yes	63	22
Unknown	1	<1
Radiotherapy regimen		
5 × 4 Gy	97	34
10 × 3/5 × 5 Gy	136	48
>40 Gy	50	18

MSCC: malignant spinal cord compression.

## Data Availability

Data of three prospective trials (NCT03070431, NCT02189473, NCT04043156) are available at clinicaltrials.gov. Otherwise, the data analyzed for this paper cannot be shared due to data protection regulations. According to the ethics committee, only evaluation of anonymized data is allowed for this study.
